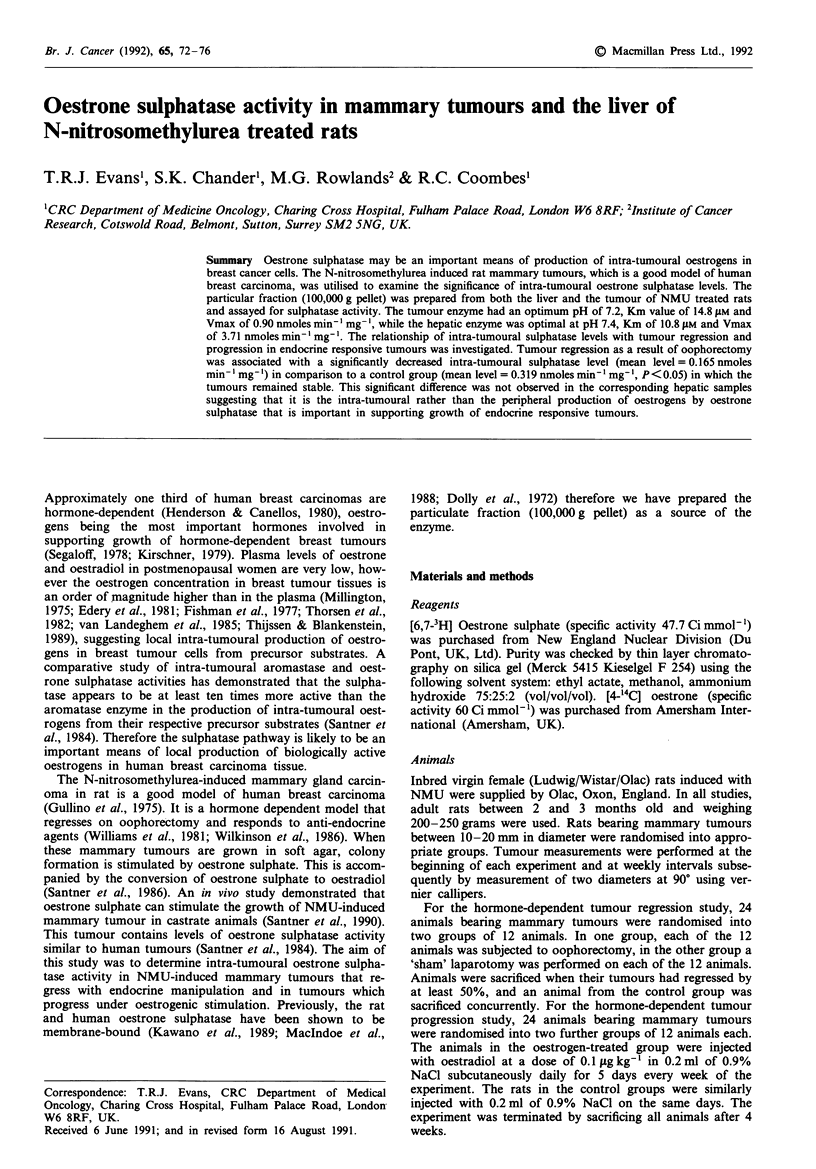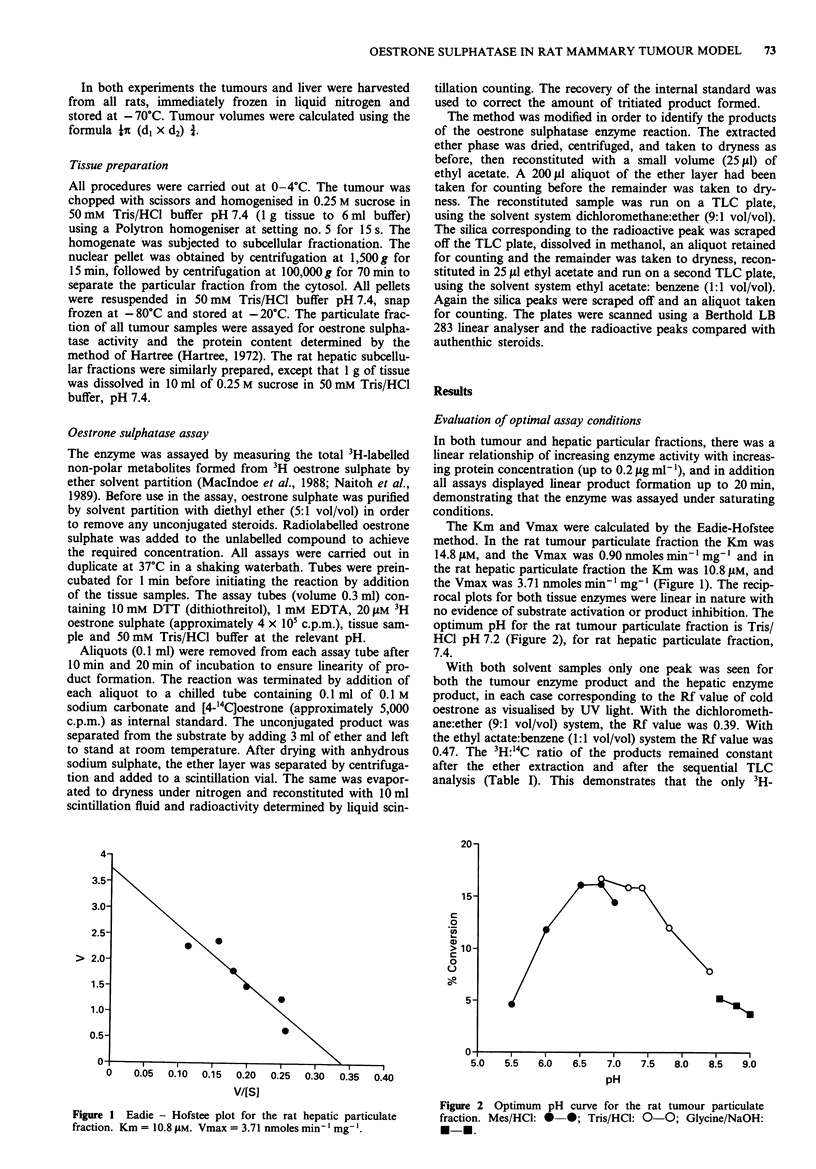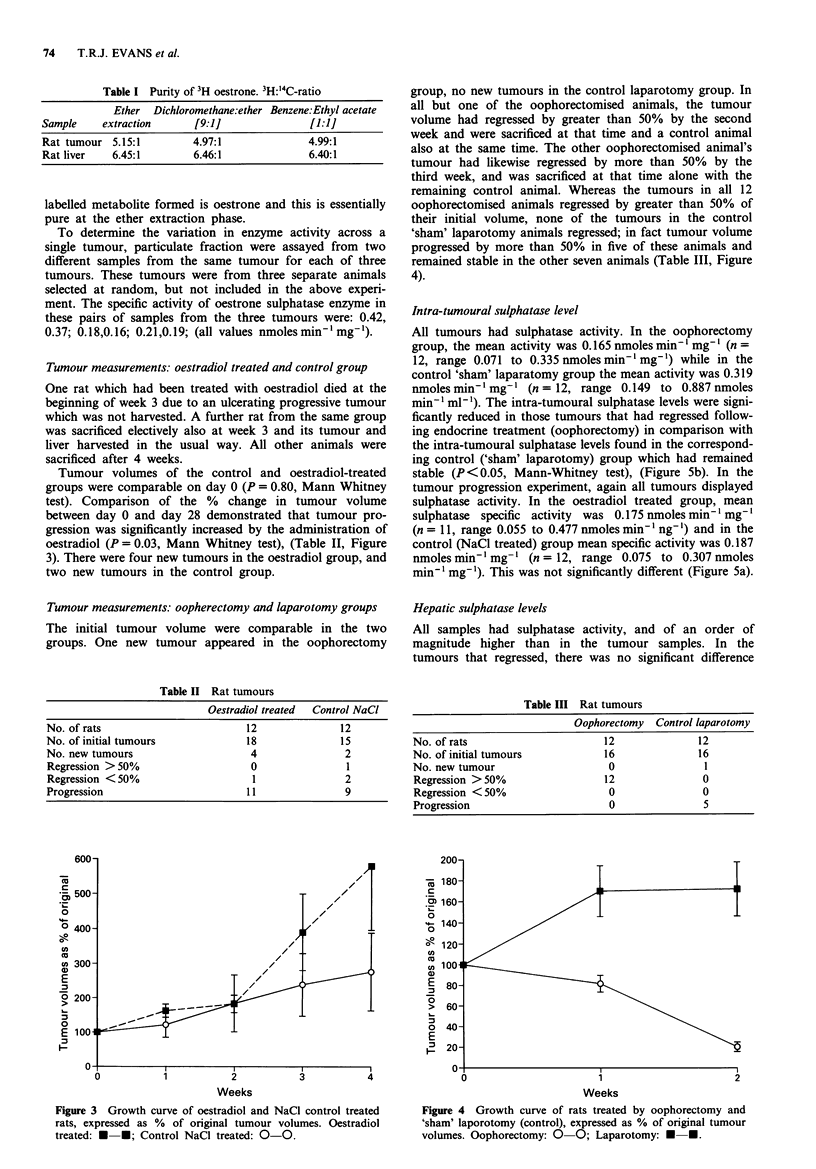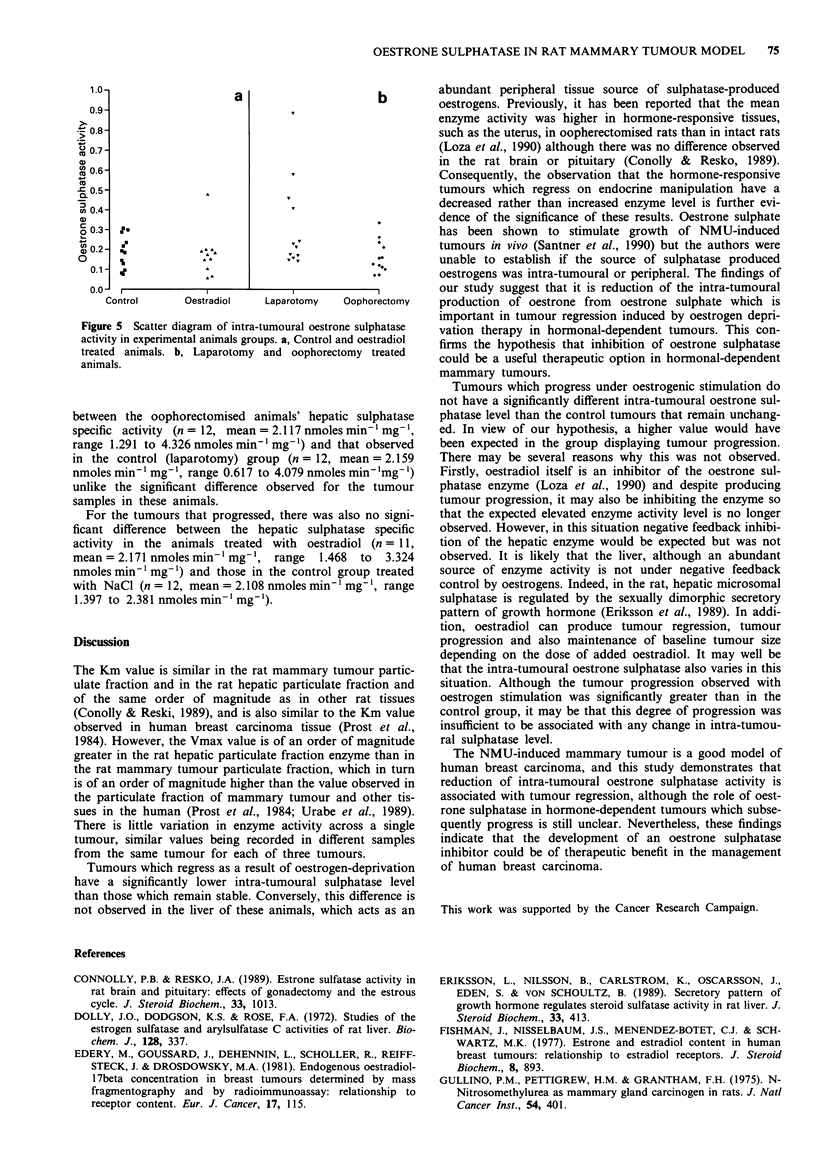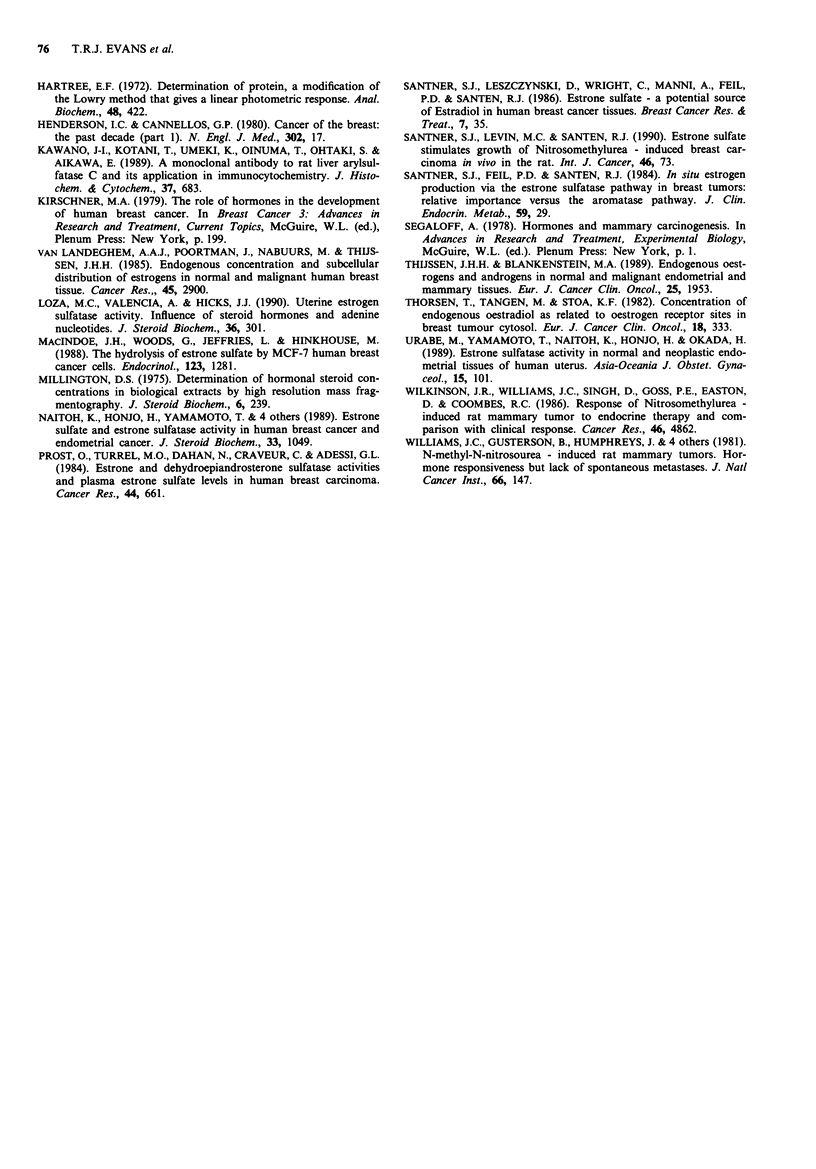# Oestrone sulphatase activity in mammary tumours and the liver of N-nitrosomethylurea treated rats.

**DOI:** 10.1038/bjc.1992.13

**Published:** 1992-01

**Authors:** T. R. Evans, S. K. Chander, M. G. Rowlands, R. C. Coombes

**Affiliations:** CRC Department of Medicine Oncology, Charing Cross Hospital, UK.

## Abstract

Oestrone sulphatase may be an important means of production of intra-tumoural oestrogens in breast cancer cells. The N-nitrosomethylurea induced rat mammary tumours, which is a good model of human breast carcinoma, was utilised to examine the significance of intra-tumoural oestrone sulphatase levels. The particular fraction (100,000 g pellet) was prepared from both the liver and the tumour of NMU treated rats and assayed for sulphatase activity. The tumour enzyme had an optimum pH of 7.2, Km value of 14.8 microM and Vmax of 0.90 nmoles min-1 mg-1, while the hepatic enzyme was optimal at pH 7.4, Km of 10.8 microM and Vmax of 3.71 nmoles min-1 mg-1. The relationship of intra-tumoural sulphatase levels with tumour regression and progression in endocrine responsive tumours was investigated. Tumour regression as a result of oophorectomy was associated with a significantly decreased intra-tumoural sulphatase level (mean level = 0.165 nmoles min-1 mg-1) in comparison to a control group (mean level = 0.319 nmoles min-1 mg-1, P less than 0.05) in which the tumours remained stable. This significant difference was not observed in the corresponding hepatic samples suggesting that it is the intra-tumoural rather than the peripheral production of oestrogens by oestrone sulphatase that is important in supporting growth of endocrine responsive tumours.


 © Macmillan Press Ltd., 1992

### Oestrone sulphatase activity in mammary tumours and the liver of N-nitrosomethylurea treated rats

T.R.J. Evans[1], S.K. Chander[1], M.G. Rowlands[2] & R.C. Coombes[1]

[1]*CRC Department of Medicine Oncology, Charing Cross Hospital, Fulham Palace Road, London W6 8RF;* [2]*Institute of Cancer Research, Cotswold Road, Belmont, Sutton, Surrey SM2 5NG, UK.*

**Summary** Oestrone sulphatase may be an important means of production of intra-tumoural oestrogens in breast cancer cells. The N-nitrosomethylurea induced rat mammary tumours, which is a good model of human breast carcinoma, was utilised to examine the significance of intra-tumoural oestrone sulphatase levels. The particular fraction (100,000 g pellet) was prepared from both the liver and the tumour of NMU treated rats and assayed for sulphatase activity. The tumour enzyme had an optimum pH of 7.2, Km value of 14.8 $\mu$M and Vmax of 0.90 nmoles $min^{-1}$ $mg^{-1}$, while the hepatic enzyme was optimal at pH 7.4, Km of 10.8 $\mu$M and Vmax of 3.71 nmoles $min^{-1}$ $mg^{-1}$. The relationship of intra-tumoural sulphatase levels with tumour regression and progression in endocrine responsive tumours was investigated. Tumour regression as a result of oophorectomy was associated with a significantly decreased intra-tumoural sulphatase level (mean level = 0.165 nmoles $min^{-1}$ $mg^{-1}$) in comparison to a control group (mean level = 0.319 nmoles $min^{-1}$ $mg^{-1}$, $P<0.05$) in which the tumours remained stable. This significant difference was not observed in the corresponding hepatic samples suggesting that it is the intra-tumoural rather than the peripheral production of oestrogens by oestrone sulphatase that is important in supporting growth of endocrine responsive tumours.

Approximately one third of human breast carcinomas are hormone-dependent (Henderson & Canellos, 1980), oestrogens being the most important hormones involved in supporting growth of hormone-dependent breast tumours (Segaloff, 1978; Kirschner, 1979). Plasma levels of oestrone and oestradiol in postmenopausal women are very low, however the oestrogen concentration in breast tumour tissues is an order of magnitude higher than in the plasma (Millington, 1975; Edery *et al.*, 1981; Fishman *et al.*, 1977; Thorsen *et al.*, 1982; van Landeghem *et al.*, 1985; Thijssen & Blankenstein, 1989), suggesting local intra-tumoural production of oestrogens in breast tumour cells from precursor substrates. A comparative study of intra-tumoural aromastase and oestrone sulphatase activities has demonstrated that the sulphatase appears to be at least ten times more active than the aromatase enzyme in the production of intra-tumoural oestrogens from their respective precursor substrates (Santner *et al.*, 1984). Therefore the sulphatase pathway is likely to be an important means of local production of biologically active oestrogens in human breast carcinoma tissue.

The N-nitrosomethylurea-induced mammary gland carcinoma in rat is a good model of human breast carcinoma (Gullino *et al.*, 1975). It is a hormone dependent model that regresses on oophorectomy and responds to anti-endocrine agents (Williams *et al.*, 1981; Wilkinson *et al.*, 1986). When these mammary tumours are grown in soft agar, colony formation is stimulated by oestrone sulphate. This is accompanied by the conversion of oestrone sulphate to oestradiol (Santner *et al.*, 1986). An *in vivo* study demonstrated that oestrone sulphate can stimulate the growth of NMU-induced mammary tumour in castrate animals (Santner *et al.*, 1990). This tumour contains levels of oestrone sulphatase activity similar to human tumours (Santner *et al.*, 1984). The aim of this study was to determine intra-tumoural oestrone sulphatase activity in NMU-induced mammary tumours that regress with endocrine manipulation and in tumours which progress under oestrogenic stimulation. Previously, the rat and human oestrone sulphatase have been shown to be membrane-bound (Kawano *et al.*, 1989; MacIndoe *et al.*, 1988; Dolly *et al.*, 1972) therefore we have prepared the particulate fraction (100,000 g pellet) as a source of the enzyme.

Correspondence: T.R.J. Evans, CRC Department of Medical Oncology, Charing Cross Hospital, Fulham Palace Road, London W6 8RF, UK.
Received 6 June 1991; and in revised form 16 August 1991.

### Materials and methods

### *Reagents*

[6,7-$^3$H] Oestrone sulphate (specific activity 47.7 Ci $mmol^{-1}$) was purchased from New England Nuclear Division (Du Pont, UK, Ltd). Purity was checked by thin layer chromatography on silica gel (Merck 5415 Kieselgel F 254) using the following solvent system: ethyl actate, methanol, ammonium hydroxide 75:25:2 (vol/vol/vol). [4-$^{14}$C] oestrone (specific activity 60 Ci $mmol^{-1}$) was purchased from Amersham International (Amersham, UK).

### *Animals*

Inbred virgin female (Ludwig/Wistar/Olac) rats induced with NMU were supplied by Olac, Oxon, England. In all studies, adult rats between 2 and 3 months old and weighing 200–250 grams were used. Rats bearing mammary tumours between 10–20 mm in diameter were randomised into appropriate groups. Tumour measurements were performed at the beginning of each experiment and at weekly intervals subsequently by measurement of two diameters at 90° using vernier callipers.

For the hormone-dependent tumour regression study, 24 animals bearing mammary tumours were randomised into two groups of 12 animals. In one group, each of the 12 animals was subjected to oophorectomy, in the other group a 'sham' laparotomy was performed on each of the 12 animals. Animals were sacrificed when their tumours had regressed by at least 50%, and an animal from the control group was sacrificed concurrently. For the hormone-dependent tumour progression study, 24 animals bearing mammary tumours were randomised into two further groups of 12 animals each. The animals in the oestrogen-treated group were injected with oestradiol at a dose of 0.1 $\mu g\ kg^{-1}$ in 0.2 ml of 0.9% NaCl subcutaneously daily for 5 days every week of the experiment. The rats in the control groups were similarly injected with 0.2 ml of 0.9% NaCl on the same days. The experiment was terminated by sacrificing all animals after 4 weeks.

In both experiments the tumours and liver were harvested from all rats, immediately frozen in liquid nitrogen and stored at −70°C. Tumour volumes were calculated using the formula $\frac{1}{6}\pi$ $(d_1 \times d_2)^{\frac{3}{4}}$.

*Tissue preparation*

All procedures were carried out at 0–4°C. The tumour was chopped with scissors and homogenised in 0.25 M sucrose in 50 mM Tris/HCl buffer pH 7.4 (1 g tissue to 6 ml buffer) using a Polytron homogeniser at setting no. 5 for 15 s. The homogenate was subjected to subcellular fractionation. The nuclear pellet was obtained by centrifugation at 1,500 *g* for 15 min, followed by centrifugation at 100,000 *g* for 70 min to separate the particular fraction from the cytosol. All pellets were resuspended in 50 mM Tris/HCl buffer pH 7.4, snap frozen at −80°C and stored at −20°C. The particulate fraction of all tumour samples were assayed for oestrone sulphatase activity and the protein content determined by the method of Hartree (Hartree, 1972). The rat hepatic subcellular fractions were similarly prepared, except that 1 g of tissue was dissolved in 10 ml of 0.25 M sucrose in 50 mM Tris/HCl buffer, pH 7.4.

*Oestrone sulphatase assay*

The enzyme was assayed by measuring the total $^3$H-labelled non-polar metabolites formed from $^3$H oestrone sulphate by ether solvent partition (MacIndoe *et al.*, 1988; Naitoh *et al.*, 1989). Before use in the assay, oestrone sulphate was purified by solvent partition with diethyl ether (5:1 vol/vol) in order to remove any unconjugated steroids. Radiolabelled oestrone sulphate was added to the unlabelled compound to achieve the required concentration. All assays were carried out in duplicate at 37°C in a shaking waterbath. Tubes were preincubated for 1 min before initiating the reaction by addition of the tissue samples. The assay tubes (volume 0.3 ml) containing 10 mM DTT (dithiothreitol), 1 mM EDTA, 20 μM $^3$H oestrone sulphate (approximately $4 \times 10^5$ c.p.m.), tissue sample and 50 mM Tris/HCl buffer at the relevant pH.

Aliquots (0.1 ml) were removed from each assay tube after 10 min and 20 min of incubation to ensure linearity of product formation. The reaction was terminated by addition of each aliquot to a chilled tube containing 0.1 ml of 0.1 M sodium carbonate and [4-$^{14}$C]oestrone (approximately 5,000 c.p.m.) as internal standard. The unconjugated product was separated from the substrate by adding 3 ml of ether and left to stand at room temperature. After drying with anhydrous sodium sulphate, the ether layer was separated by centrifugation and added to a scintillation vial. The same was evaporated to dryness under nitrogen and reconstituted with 10 ml scintillation fluid and radioactivity determined by liquid scintillation counting. The recovery of the internal standard was used to correct the amount of tritiated product formed.

The method was modified in order to identify the products of the oestrone sulphatase enzyme reaction. The extracted ether phase was dried, centrifuged, and taken to dryness as before, then reconstituted with a small volume (25 μl) of ethyl acetate. A 200 μl aliquot of the ether layer had been taken for counting before the remainder was taken to dryness. The reconstituted sample was run on a TLC plate, using the solvent system dichloromethane:ether (9:1 vol/vol). The silica corresponding to the radioactive peak was scraped off the TLC plate, dissolved in methanol, an aliquot retained for counting and the remainder was taken to dryness, reconstituted in 25 μl ethyl acetate and run on a second TLC plate, using the solvent system ethyl acetate: benzene (1:1 vol/vol). Again the silica peaks were scraped off and an aliquot taken for counting. The plates were scanned using a Berthold LB 283 linear analyser and the radioactive peaks compared with authenthic steroids.

### Results

*Evaluation of optimal assay conditions*

In both tumour and hepatic particular fractions, there was a linear relationship of increasing enzyme activity with increasing protein concentration (up to 0.2 μg ml$^{-1}$), and in addition all assays displayed linear product formation up to 20 min, demonstrating that the enzyme was assayed under saturating conditions.

The Km and Vmax were calculated by the Eadie-Hofstee method. In the rat tumour particulate fraction the Km was 14.8 μM, and the Vmax was 0.90 nmoles min$^{-1}$ mg$^{-1}$ and in the rat hepatic particulate fraction the Km was 10.8 μM, and the Vmax was 3.71 nmoles min$^{-1}$ mg$^{-1}$ (Figure 1). The reciprocal plots for both tissue enzymes were linear in nature with no evidence of substrate activation or product inhibition. The optimum pH for the rat tumour particulate fraction is Tris/HCl pH 7.2 (Figure 2), for rat hepatic particulate fraction, 7.4.

With both solvent samples only one peak was seen for both the tumour enzyme product and the hepatic enzyme product, in each case corresponding to the Rf value of cold oestrone as visualised by UV light. With the dichloromethane:ether (9:1 vol/vol) system, the Rf value was 0.39. With the ethyl actate:benzene (1:1 vol/vol) system the Rf value was 0.47. The $^3$H:$^{14}$C ratio of the products remained constant after the ether extraction and after the sequential TLC analysis (Table I). This demonstrates that the only $^3$H-

**Figure 1** Eadie – Hofstee plot for the rat hepatic particulate fraction. Km = 10.8 μM. Vmax = 3.71 nmoles min$^{-1}$ mg$^{-1}$.

**Figure 2** Optimum pH curve for the rat tumour particulate fraction. Mes/HCl: ●—●; Tris/HCl: O—O; Glycine/NaOH: ■—■.

**Table I** Purity of $^{3}H$ oestrone. $^{3}H$:$^{14}C$-ratio

| Sample | Ether extraction | Dichloromethane:ether [9:1] | Benzene:Ethyl acetate [1:1] |
|---|---|---|---|
| Rat tumour | 5.15:1 | 4.97:1 | 4.99:1 |
| Rat liver | 6.45:1 | 6.46:1 | 6.40:1 |

labelled metabolite formed is oestrone and this is essentially pure at the ether extraction phase.

To determine the variation in enzyme activity across a single tumour, particulate fraction were assayed from two different samples from the same tumour for each of three tumours. These tumours were from three separate animals selected at random, but not included in the above experiment. The specific activity of oestrone sulphatase enzyme in these pairs of samples from the three tumours were: 0.42, 0.37; 0.18,0.16; 0.21,0.19; (all values nmoles $min^{-1}$ $mg^{-1}$).

*Tumour measurements: oestradiol treated and control group*

One rat which had been treated with oestradiol died at the beginning of week 3 due to an ulcerating progressive tumour which was not harvested. A further rat from the same group was sacrificed electively also at week 3 and its tumour and liver harvested in the usual way. All other animals were sacrificed after 4 weeks.

Tumour volumes of the control and oestradiol-treated groups were comparable on day 0 ($P = 0.80$, Mann Whitney test). Comparison of the % change in tumour volume between day 0 and day 28 demonstrated that tumour progression was significantly increased by the administration of oestradiol ($P = 0.03$, Mann Whitney test), (Table II, Figure 3). There were four new tumours in the oestradiol group, and two new tumours in the control group.

*Tumour measurements: oopherectomy and laparotomy groups*

The initial tumour volume were comparable in the two groups. One new tumour appeared in the oophorectomy group, no new tumours in the control laparotomy group. In all but one of the oophorectomised animals, the tumour volume had regressed by greater than 50% by the second week and were sacrificed at that time and a control animal also at the same time. The other oophorectomised animal's tumour had likewise regressed by more than 50% by the third week, and was sacrificed at that time alone with the remaining control animal. Whereas the tumours in all 12 oophorectomised animals regressed by greater than 50% of their initial volume, none of the tumours in the control 'sham' laparotomy animals regressed; in fact tumour volume progressed by more than 50% in five of these animals and remained stable in the other seven animals (Table III, Figure 4).

*Intra-tumoural sulphatase level*

All tumours had sulphatase activity. In the oophorectomy group, the mean activity was 0.165 nmoles $min^{-1}$ $mg^{-1}$ ($n = 12$, range 0.071 to 0.335 nmoles $min^{-1}$ $mg^{-1}$) while in the control 'sham' laparatomy group the mean activity was 0.319 nmoles $min^{-1}$ $mg^{-1}$ ($n = 12$, range 0.149 to 0.887 nmoles $min^{-1}$ $ml^{-1}$). The intra-tumoural sulphatase levels were significantly reduced in those tumours that had regressed following endocrine treatment (oophorectomy) in comparison with the intra-tumoural sulphatase levels found in the corresponding control ('sham' laparotomy) group which had remained stable ($P < 0.05$, Mann-Whitney test), (Figure 5b). In the tumour progression experiment, again all tumours displayed sulphatase activity. In the oestradiol treated group, mean sulphatase specific activity was 0.175 nmoles $min^{-1}$ $mg^{-1}$ ($n = 11$, range 0.055 to 0.477 nmoles $min^{-1}$ $ng^{-1}$) and in the control (NaCl treated) group mean specific activity was 0.187 nmoles $min^{-1}$ $mg^{-1}$ ($n = 12$, range 0.075 to 0.307 nmoles $min^{-1}$ $mg^{-1}$). This was not significantly different (Figure 5a).

*Hepatic sulphatase levels*

All samples had sulphatase activity, and of an order of magnitude higher than in the tumour samples. In the tumours that regressed, there was no significant difference

**Table II** Rat tumours

| | Oestradiol treated | Control NaCl |
|---|---|---|
| No. of rats | 12 | 12 |
| No. of initial tumours | 18 | 15 |
| No. new tumours | 4 | 2 |
| Regression >50% | 0 | 1 |
| Regression <50% | 1 | 2 |
| Progression | 11 | 9 |

**Table III** Rat tumours

| | Oophorectomy | Control laparotomy |
|---|---|---|
| No. of rats | 12 | 12 |
| No. of initial tumours | 16 | 16 |
| No. new tumour | 0 | 1 |
| Regression >50% | 12 | 0 |
| Regression <50% | 0 | 0 |
| Progression | 0 | 5 |

**Figure 3** Growth curve of oestradiol and NaCl control treated rats, expressed as % of original tumour volumes. Oestradiol treated: ■—■; Control NaCl treated: O—O.

**Figure 4** Growth curve of rats treated by oophorectomy and 'sham' laporotomy (control), expressed as % of original tumour volumes. Oophorectomy: O—O; Laparotomy: ■—■.

**Figure 5** Scatter diagram of intra-tumoural oestrone sulphatase activity in experimental animals groups. **a**, Control and oestradiol treated animals. **b**, Laparotomy and oophorectomy treated animals.

between the oophorectomised animals' hepatic sulphatase specific activity ($n = 12$, mean = 2.117 nmoles $min^{-1}$ $mg^{-1}$, range 1.291 to 4.326 nmoles $min^{-1}$ $mg^{-1}$) and that observed in the control (laparotomy) group ($n = 12$, mean = 2.159 nmoles $min^{-1}$ $mg^{-1}$, range 0.617 to 4.079 nmoles $min^{-1}mg^{-1}$) unlike the significant difference observed for the tumour samples in these animals.

For the tumours that progressed, there was also no significant difference between the hepatic sulphatase specific activity in the animals treated with oestradiol ($n = 11$, mean = 2.171 nmoles $min^{-1}$ $mg^{-1}$, range 1.468 to 3.324 nmoles $min^{-1}$ $mg^{-1}$) and those in the control group treated with NaCl ($n = 12$, mean = 2.108 nmoles $min^{-1}$ $mg^{-1}$, range 1.397 to 2.381 nmoles $min^{-1}$ $mg^{-1}$).

### Discussion

The Km value is similar in the rat mammary tumour particulate fraction and in the rat hepatic particulate fraction and of the same order of magnitude as in other rat tissues (Conolly & Reski, 1989), and is also similar to the Km value observed in human breast carcinoma tissue (Prost *et al.*, 1984). However, the Vmax value is of an order of magnitude greater in the rat hepatic particulate fraction enzyme than in the rat mammary tumour particulate fraction, which in turn is of an order of magnitude higher than the value observed in the particulate fraction of mammary tumour and other tissues in the human (Prost *et al.*, 1984; Urabe *et al.*, 1989). There is little variation in enzyme activity across a single tumour, similar values being recorded in different samples from the same tumour for each of three tumours.

Tumours which regress as a result of oestrogen-deprivation have a significantly lower intra-tumoural sulphatase level than those which remain stable. Conversely, this difference is not observed in the liver of these animals, which acts as an abundant peripheral tissue source of sulphatase-produced oestrogens. Previously, it has been reported that the mean enzyme activity was higher in hormone-responsive tissues, such as the uterus, in oopherectomised rats than in intact rats (Loza *et al.*, 1990) although there was no difference observed in the rat brain or pituitary (Conolly & Resko, 1989). Consequently, the observation that the hormone-responsive tumours which regress on endocrine manipulation have a decreased rather than increased enzyme level is further evidence of the significance of these results. Oestrone sulphate has been shown to stimulate growth of NMU-induced tumours *in vivo* (Santner *et al.*, 1990) but the authors were unable to establish if the source of sulphatase produced oestrogens was intra-tumoural or peripheral. The findings of our study suggest that it is reduction of the intra-tumoural production of oestrone from oestrone sulphate which is important in tumour regression induced by oestrogen deprivation therapy in hormonal-dependent tumours. This confirms the hypothesis that inhibition of oestrone sulphatase could be a useful therapeutic option in hormonal-dependent mammary tumours.

Tumours which progress under oestrogenic stimulation do not have a significantly different intra-tumoural oestrone sulphatase level than the control tumours that remain unchanged. In view of our hypothesis, a higher value would have been expected in the group displaying tumour progression. There may be several reasons why this was not observed. Firstly, oestradiol itself is an inhibitor of the oestrone sulphatase enzyme (Loza *et al.*, 1990) and despite producing tumour progression, it may also be inhibiting the enzyme so that the expected elevated enzyme activity level is no longer observed. However, in this situation negative feedback inhibition of the hepatic enzyme would be expected but was not observed. It is likely that the liver, although an abundant source of enzyme activity is not under negative feedback control by oestrogens. Indeed, in the rat, hepatic microsomal sulphatase is regulated by the sexually dimorphic secretory pattern of growth hormone (Eriksson *et al.*, 1989). In addition, oestradiol can produce tumour regression, tumour progression and also maintenance of baseline tumour size depending on the dose of added oestradiol. It may well be that the intra-tumoural oestrone sulphatase also varies in this situation. Although the tumour progression observed with oestrogen stimulation was significantly greater than in the control group, it may be that this degree of progression was insufficient to be associated with any change in intra-tumoural sulphatase level.

The NMU-induced mammary tumour is a good model of human breast carcinoma, and this study demonstrates that reduction of intra-tumoural oestrone sulphatase activity is associated with tumour regression, although the role of oestrone sulphatase in hormone-dependent tumours which subsequently progress is still unclear. Nevertheless, these findings indicate that the development of an oestrone sulphatase inhibitor could be of therapeutic benefit in the management of human breast carcinoma.

This work was supported by the Cancer Research Campaign.

### References

CONNOLLY, P.B. & RESKO, J.A. (1989). Estrone sulfatase activity in rat brain and pituitary: effects of gonadectomy and the estrous cycle. *J. Steroid Biochem.*, **33**, 1013.

DOLLY, J.O., DODGSON, K.S. & ROSE, F.A. (1972). Studies of the estrogen sulfatase and arylsulfatase C activities of rat liver. *Biochem. J.*, **128**, 337.

EDERY, M., GOUSSARD, J., DEHENNIN, L., SCHOLLER, R., REIFFSTECK, J. & DROSDOWSKY, M.A. (1981). Endogenous oestradiol-17beta concentration in breast tumours determined by mass fragmentography and by radioimmunoassay: relationship to receptor content. *Eur. J. Cancer*, **17**, 115.

ERIKSSON, L., NILSSON, B., CARLSTROM, K., OSCARSSON, J., EDEN, S. & VON SCHOULTZ, B. (1989). Secretory pattern of growth hormone regulates steroid sulfatase activity in rat liver. *J. Steroid Biochem.*, **33**, 413.

FISHMAN, J., NISSELBAUM, J.S., MENENDEZ-BOTET, C.J. & SCHWARTZ, M.K. (1977). Estrone and estradiol content in human breast tumours: relationship to estradiol receptors. *J. Steroid Biochem.*, **8**, 893.

GULLINO, P.M., PETTIGREW, H.M. & GRANTHAM, F.H. (1975). N-Nitrosomethylurea as mammary gland carcinogen in rats. *J. Natl Cancer Inst.*, **54**, 401.

HARTREE, E.F. (1972). Determination of protein, a modification of the Lowry method that gives a linear photometric response. *Anal. Biochem.*, **48**, 422.

HENDERSON, I.C. & CANNELLOS, G.P. (1980). Cancer of the breast: the past decade (part 1). *N. Engl. J. Med.*, **302**, 17.

KAWANO, J-I., KOTANI, T., UMEKI, K., OINUMA, T., OHTAKI, S. & AIKAWA, E. (1989). A monoclonal antibody to rat liver arylsulfatase C and its application in immunocytochemistry. *J. Histochem. & Cytochem.*, **37**, 683.

KIRSCHNER, M.A. (1979). The role of hormones in the development of human breast cancer. In *Breast Cancer 3: Advances in Research and Treatment, Current Topics*, McGuire, W.L. (ed.), Plenum Press: New York, p. 199.

VAN LANDEGHEM, A.A.J., POORTMAN, J., NABUURS, M. & THIJSSEN, J.H.H. (1985). Endogenous concentration and subcellular distribution of estrogens in normal and malignant human breast tissue. *Cancer Res.*,, **45**, 2900.

LOZA, M.C., VALENCIA, A. & HICKS, J.J. (1990). Uterine estrogen sulfatase activity. Influence of steroid hormones and adenine nucleotides. *J. Steroid Biochem.*, **36**, 301.

MACINDOE, J.H., WOODS, G., JEFFRIES, L. & HINKHOUSE, M. (1988). The hydrolysis of estrone sulfate by MCF-7 human breast cancer cells. *Endocrinol.*, **123**, 1281.

MILLINGTON, D.S. (1975). Determination of hormonal steroid concentrations in biological extracts by high resolution mass fragmentography. *J. Steroid Biochem.*, **6**, 239.

NAITOH, K., HONJO, H., YAMAMOTO, T. & 4 others (1989). Estrone sulfate and estrone sulfatase activity in human breast cancer and endometrial cancer. *J. Steroid Biochem.*, **33**, 1049.

PROST, O., TURREL, M.O., DAHAN, N., CRAVEUR, C. & ADESSI, G.L. (1984). Estrone and dehydroepiandrosterone sulfatase activities and plasma estrone sulfate levels in human breast carcinoma. *Cancer Res.*, **44**, 661.

SANTNER, S.J., LESZCZYNSKI, D., WRIGHT, C., MANNI, A., FEIL, P.D. & SANTEN, R.J. (1986). Estrone sulfate - a potential source of Estradiol in human breast cancer tissues. *Breast Cancer Res. & Treat.*, **7**, 35.

SANTNER, S.J., LEVIN, M.C. & SANTEN, R.J. (1990). Estrone sulfate stimulates growth of Nitrosomethylurea - induced breast carcinoma *in vivo* in the rat. *Int. J. Cancer*, **46**, 73.

SANTNER, S.J., FEIL, P.D. & SANTEN, R.J. (1984). *In situ* estrogen production via the estrone sulfatase pathway in breast tumors: relative importance versus the aromatase pathway. *J. Clin. Endocrin. Metab.*, **59**, 29.

SEGALOFF, A. (1978). Hormones and mammary carcinogenesis. In *Advances in Research and Treatment, Experimental Biology*, McGuire, W.L. (ed.). Plenum Press: New York, p. 1.

THIJSSEN, J.H.H. & BLANKENSTEIN, M.A. (1989). Endogenous oestrogens and androgens in normal and malignant endometrial and mammary tissues. *Eur. J. Cancer Clin. Oncol.*, **25**, 1953.

THORSEN, T., TANGEN, M. & STOA, K.F. (1982). Concentration of endogenous oestradiol as related to oestrogen receptor sites in breast tumour cytosol. *Eur. J. Cancer Clin. Oncol.*, **18**, 333.

URABE, M., YAMAMOTO, T., NAITOH, K., HONJO, H. & OKADA, H. (1989). Estrone sulfatase activity in normal and neoplastic endometrial tissues of human uterus. *Asia-Oceania J. Obstet. Gynaceol.*, **15**, 101.

WILKINSON, J.R., WILLIAMS, J.C., SINGH, D., GOSS, P.E., EASTON, D. & COOMBES, R.C. (1986). Response of Nitrosomethylurea - induced rat mammary tumor to endocrine therapy and comparison with clinical response. *Cancer Res.*, **46**, 4862.

WILLIAMS, J.C., GUSTERSON, B., HUMPHREYS, J. & 4 others (1981). N-methyl-N-nitrosourea - induced rat mammary tumors. Hormone responsiveness but lack of spontaneous metastases. *J. Natl Cancer Inst.*, **66**, 147.